# *DPYD* and *UGT1A1* Genotype‐Based Dosing for Fluoropyrimidines and Irinotecan Chemotherapy: Variant‐Specific Impact on Treatment Intensity and Toxicity

**DOI:** 10.1002/ijc.70580

**Published:** 2026-06-09

**Authors:** Martina Gambron, Elena Peruzzi, Samantha Perfler, Marcella Montico, Riccardo Cecchin, Elena De Mattia, Michele Spina, Camilla Lisanti, Serena Corsetti, Luisa Foltran, Fabio Puglisi, Rossana Roncato, Erika Cecchin

**Affiliations:** ^1^ Experimental and Clinical Pharmacology Unit Centro di Riferimento Oncologico di Aviano (CRO), IRCCS Aviano Italy; ^2^ Scientific Directorate Centro di Riferimento Oncologico di Aviano (CRO), IRCCS Aviano Italy; ^3^ Division of Medical Oncology and Immune‐Related Tumors Centro di Riferimento Oncologico di Aviano (CRO), IRCCS Aviano Italy; ^4^ Department of Medical Oncology Centro di Riferimento Oncologico di Aviano (CRO), IRCCS Aviano Italy; ^5^ Department of Medicine (DMED) University of Udine Udine Italy

**Keywords:** dose intensity, *DPYD*, pharmacogenetic testing, precision dosing, *UGT1A1*

## Abstract

*DPYD* and *UGT1A1* variants significantly affect fluoropyrimidines and irinotecan safety. While genotype‐driven dosing reduces severe toxicity, the impact of individual variants remains incompletely investigated. This retrospective cohort study includes 178 cancer patients with a matched actionable *DPYD* and/or *UGT1A1* genotypes, defined by the presence of guideline‐recommended variants (*DPYD*: rs3918290, DPYD*2A, c.1905+1G>A; rs55886062, DPYD*13, c.1679T>G; rs67376798, c.2846A>T; rs56038477, c.1236G>A (HapB3); *UGT1A1*: rs8175347, UGT1A1*28/*37 and rs4148323, UGT1A1*6, c.211G>A), and treated with fluoropyrimidines and/or irinotecan. Patients were classified into standard‐of‐care (posttreatment genotyping; *n* = 97) or genotype‐driven group (pretreatment genotyping with DPWG‐driven dosing; *n* = 81). Clinically relevant toxicity and relative dose intensity (RDI), a proxy for drug exposure, were evaluated by genotype. Genotype‐driven dosing significantly reduced overall clinically relevant toxicity (16.0% vs. 43.3%; *p* < 0.001). Among *DPYD* c.1905+1G>A heterozygous carriers, clinically relevant toxicities occurred only within standard‐of‐care (61.5% vs. 0%, *p* = 0.007), with lower RDI despite full starting dose (41% [IQR 13–62] vs. 48% [IQR 46–50], *p* = 0.425). In *DPYD* c.2846A>T heterozygous carriers, standard‐of‐care had slightly higher RDI (63% [IQR 54–80] vs. 50% [IQR 43–50], *p* = 0.007) but doubled toxicity rate (27% vs. 67%, *p* = 0.092). Conversely, *DPYD* c.1236G>A (HapB3) heterozygous carriers experienced similarly low toxicity rates (23.7 vs. 13.2, *p* = 0.375), but genotype‐driven dosing markedly reduced RDI (50% [IQR 43–55] vs. 82% [IQR 63–92], *p* < 0.001). In UGT1A1*28 homozygous carriers, genotype‐driven dosing reduced toxicity (21.1% vs. 45.7%, *p* = 0.086) while preserving comparable RDI (58% [IQR 50–69] vs. 61% [IQR 41–76], *p* = 0.906). These findings suggest uniform genotype‐based strategies may not fully capture variant‐specific effects, particularly for *DPYD* c.1236G>A (HapB3), supporting more flexible dosing approaches.

## What's New?

Pre‐treatment *DPYD* and *UGT1A1* genotyping is increasingly used to prevent fluoropyrimidine‐ and irinotecan‐related toxicity, but variant‐specific real‐world effects remain unclear. In an unselected cohort of cancer patients with actionable genotypes, genotype‐driven dosing improved safety while preserving treatment exposure in high‐risk *DPYD* c.1905+1G>A and UGT1A1*28 carriers. In *DPYD* c.2846A>T carriers, it substantially reduced toxicity burden, with only slightly lower RDI than standard‐of‐care. Conversely, in *DPYD* c.1236G>A/HapB3 heterozygous carriers, genotype‐driven dosing did not show a clear toxicity benefit and may lead to reduced treatment exposure supporting a more flexible, adaptive dosing strategies.

## Graphical abstract

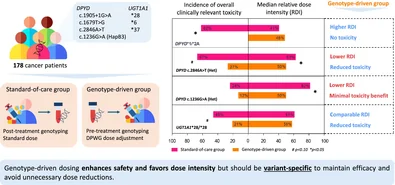

**Abbreviations**
5‐FU: 5‐fluorouracil
Cape: capecitabine
CPIC: The Clinical Pharmacogenetics Implementation Consortium
CRO: Centro di Riferimento Oncologico
CTCAE: Common Terminology Criteria for Adverse Events
DPD: dihydropyrimidine dehydrogenase
DPWG: Dutch Pharmacogenetics Working Group
DPYD: gene encoding DPD
EMA: European Medicines Agency
FDA: US Food and Drug Administration
FL: fluoropyrimidines
HapB3: *DPYD* haplotype tagged by c.1129–5923C>G
IQR: interquartile range
IRCCS: Istituto di Ricovero e Cura a Carattere Scientifico
IRI: irinotecan
Pt: platinum agent
RDI: relative dose intensity
RT: radiotherapy
SNP: single nucleotide polymorphism
UGT1A1: UDP‐glucuronosyltransferase 1A1
VNTR: variable number tandem repeat

## 1 Introduction

Fluoropyrimidines, including 5‐fluorouracil (5‐FU) and capecitabine, remain widely used in the treatment of a broad range of solid tumors, such as gastrointestinal, breast, and head and neck cancers [1]. Although generally well tolerated, serious adverse drug reactions occur in approximately 20%–30% of patients. In advanced‐stage disease, fluoropyrimidines are frequently combined with other cytotoxic agents, such as oxaliplatin or irinotecan, and often co‐administered with targeted biologic therapies like cetuximab or bevacizumab [2, 3, 4]. Particularly, regimens combining fluoropyrimidines and irinotecan have resulted in significant improvements in treatment outcomes and overall survival; however, these benefits are accompanied by an increased toxicity burden due to overlapping gastrointestinal and hematologic toxicities [5].

Genetic variants in *DPYD* gene, causing dihydropyrimidine dehydrogenase (DPD) deficiency, have been associated with an increased risk of severe toxicity following fluoropyrimidine‐based chemotherapy. Similarly, a reduced activity of the UDP‐glucuronosyltransferase 1A1 enzyme (UGT1A1) has been linked to a higher incidence of toxicity, particularly neutropenia and diarrhea, in patients treated with irinotecan [6, 7]. Pretreatment genotyping of clinically relevant *DPYD* variants, rs3918290 (DPYD*2A, c.1905+1G>A), rs55886062 (DPYD*13, c.1679T>G), rs67376798 (c.2846A>T), and rs56038477 (c.1236G>A; tagging SNP for the causative variant rs75017182, c.1129–5923C>G), is currently recommended by regulatory agencies, including EMA and FDA [8, 9]. For *UGT1A1*, variants most strongly associated with increased toxicity risk include the decreased function variants: rs8175347 (UGT1A1*28 or UGT1A1*37) and rs4148323 (UGT1A1*6, c.211G>A).

Current pharmacogenetic guidelines from the Dutch Pharmacogenetics Working Group (DPWG) recommend a flat dose reduction of 50% for patients with *DPYD* gene activity score of 1.0 (rs3918290 and rs55886062 heterozygous variant carriers) and 1.5 (rs67376798 and rs56038477 heterozygous variant carriers) [10]. Similarly, for patients carrying two decreased‐function *UGT1A1* alleles (poor metabolizers), a 30% dose reduction for irinotecan is recommended [11]. Pharmacogenetic‐driven dosing based on *DPYD* and *UGT1A1* has been increasingly implemented in clinical practice across many countries [12, 13], supported by recent evidence suggesting that prospective dose reductions do not compromise drug exposure or overall survival [14, 15]. Nevertheless, concerns about potential underexposure, especially for specific genetic variants at low functional impact, such as HapB3, continue to act as a barrier to broader implementation [16].

A secondary analysis of *DPYD* and *UGT1A1* variants carriers treated with fluoropyrimidine and/or irinotecan within the PREPARE trial showed that genotype‐based dose reductions did not significantly affect treatment intensity overall, but the small sample size prevented the possibility to evaluate any variant‐specific effect [17]. More recently, additional studies have further investigated the impact of *DPYD* genotype‐driven dosing on drug exposure, demonstrating that dose reductions should ideally be tailored according to the specific *DPYD* variant [18, 19]. However, these studies lacked a comparison with a standard‐of‐care group.

The primary aim of this study was to comparatively assess clinically validated *DPYD* and *UGT1A1* variants with respect to treatment‐related toxicities and relative dose intensity (RDI), and to investigate how actionable genotypes translate into treatment modifications and differences in treatment intensity.

## 2 Material and Methods

### 2.1 Study Design and Population

The study was designed as a retrospective cohort study comparing two non‐contemporaneous groups of patients treated in different time periods, reflecting the historical evolution of pharmacogenetic‐driven dose adjustments at our institution. All patients included in the study were selected from a prospective patients' collection of 4556 clinical cases enrolled in different clinical protocols coordinated by the Experimental and Clinical Pharmacology Unit of Centro di Riferimento Oncologico di Aviano (CRO), IRCCS [7, 20, 21, 22]. Patients were considered eligible based on the following criteria: (i) fluoropyrimidines and/or irinotecan‐based chemotherapy; (ii) presence of an actionable genotype, defined by both (a) carriage of at least one guideline‐recommended *DPYD* variant (rs3918290, rs55886062, rs67376798, or rs56038477 tagging SNP for HapB3) or two decreased‐function *UGT1A1* alleles (rs8175347 or rs4148323), and (b) exposure to the “matched” actionable drug. For this study, a “matched” actionable treatment was defined as a therapeutic regimen corresponding to the patient's pharmacogenetic variant (i.e., *DPYD* variant carriers treated with fluoropyrimidines and *UGT1A1* variant carriers treated with irinotecan); (iii) complete clinical data, including toxicity follow‐up for the entire duration of treatment; (iv) signed informed consent approved by the local Ethical Committee for the use of data for research purposes.

**FIGURE 1:**
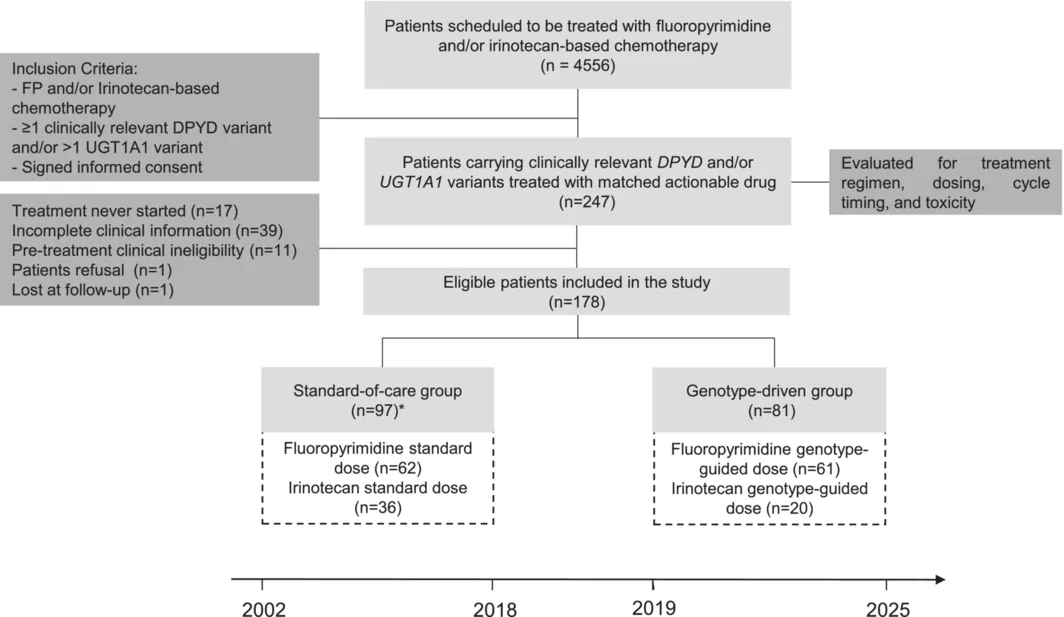
Flow chart of study design and patient selection. Reasons for patient exclusion are detailed in the diagram. *In the standard‐of‐care group one patient carried variants in both genes; therefore, she was considered in both the fluoropyrimidine and irinotecan treatment groups. FP, Fluoropyrimidines.

Exclusion criteria included: (i) pretreatment clinical ineligibility; (ii) incomplete clinical information or follow‐up; (iii) treatment planned but not initiated or early patient withdrawal. (Figure 1).

The two primary endpoints of the study were: (i) clinically relevant toxicity related to fluoropyrimidine and/or irinotecan, defined as grade ≥ 4 hematologic toxicity or grade ≥ 3 non‐hematologic toxicity; (ii) fluoropyrimidines and irinotecan RDI.

### 2.2 Clinical Data Collection

Clinical and demographic data were already available and prospectively collected within previously conducted clinical protocols [7, 20, 21, 22]. Additional information was retrospectively extracted from patients’ medical records for the purpose of this analysis. Data were collected at two levels: patient‐specific (actual treatment delivered) and planned treatment characteristics (reference standard). Patient‐specific data included: (i) genotype; (ii) number of administered cycles; (iii) delivered dose (mg) per administration; (iv) treatment duration, including cycle length; (v) treatment delays and temporary interruptions; (vi) number of days of suspension; (vii) reasons for treatment discontinuation; (viii) toxicity data. Planned treatment characteristics included: (i) treatment setting and regimen; (ii) intended number of cycles and cycle length according to standard protocols; (iii) dosing schedule.

### 2.3 Toxicity Data and RDI Calculation

Toxicity data were classified according to National Cancer Institute Common Terminology Criteria for Adverse Events (CTCAE), version 5.0. For each chemotherapy cycle, severity grade and onset date of adverse events were annotated. Data on specific toxicity subtypes (neutropenia, leukopenia, diarrhea, mucositis, hand‐foot syndrome) were evaluated individually. Toxicity was classified as “acute” if developed during the first treatment cycle, or “overall” if evaluated over the entire duration of treatment. Toxicity was monitored throughout the entire duration of treatment and systematically recorded on a per‐cycle basis.

Dose modifications were defined as a reduction or escalation of at least 15% of the standard dose or of the previous dose received. RDI was calculated as the overall amount of drug received (milligrams) by unit of time (considering dose modifications, treatment delays, and treatment interruptions), over the overall amount of drug planned, based on the standard chemotherapy scheme. RDI was expressed as a percentage and evaluated both during the first treatment cycle and over the entire duration of treatment. Body weight changes were accounted for in the RDI calculation at each cycle.

### 2.4 Genotyping Data

Genotyping results were retrieved from existing pharmacogenetic reports and used for the present study; no additional laboratory analysis was performed specifically for this work. Briefly, genotyping was previously performed by TaqMan assays and/or pyrosequencing for detecting single nucleotide variants. For *UGT1A1* rs8175347 (UGT1A1*28 or *37), a fragment analysis assay on an automated Sanger sequencer was performed to determine the number of tandem repeats (VNTRs) specifically associated with each variant. For *DPYD*, the HapB3 haplotype was inferred using the tagging single nucleotide polymorphism rs56038477 (c.1236G>A), as the causal intronic variant rs75017182 (c.1129‐5923C>G) was not directly assessed. For patients classified as “genotype‐driven group,” the pharmacogenetic results, including both the patient's genotype and the dosing recommendation according to the most recent version of the pharmacogenetics guidelines available [10, 11], were delivered to the treating physician before treatment. For patients classified as “standard‐of‐care group,” the pharmacogenetic results were obtained after treatment and were not used to tailor drug dosage.

### 2.5 Statistical Analysis

Data are presented as absolute frequencies and percentages or as medians and interquartile ranges (IQRs). Differences between categorical variables were assessed using Chi‐squared or Fisher's exact test as appropriate, whereas differences between continuous variables were evaluated based on median values using the Mann–Whitney test. When the number of observations allows, multivariate logistic regression was carried out to adjust for potential confounders such as sex and age. Data are reported as adjusted Odds Ratio (adj OR) and relative 95% Confidence Interval (95% CI). Analyses and graphs were performed using GraphPad Prism version 8.0.1 and Stata 14.2.

## 3 Results

### 3.1 Study Population Characteristics and Genotypes

A total of 178 eligible patients were included, with 97 (54.5%) included in the standard‐of‐care group and 81 (45.5%) in the genotype‐driven dosing group. Median age was 65 years (IQR 56–71); sex and ethnicity distribution were comparable between groups (Table 1).

**TABLE 1:** Patient demographic and clinical characteristics.

	Overall population		Standard‐of‐care		Genotype‐driven		*p* ^*^
Eligible patients	*n*	%	*n*	%	*n*	%	
Number of patients	178		97	54.5	81	45.5	—
Age, years median (IQR)	65	56–71	64	54–69	67	59–73	—
Female	70	39.3	39	40.2	31	38.3	0.888
Ethnicity							
Caucasian	171	96.1	92	94.8	79	97.5	0.457
Other^a^	7	3.9	5	5.2	2	2.5	
Heterozygous *DPYD* variant carriers^b^							
*DPYD* c.1905+1G>A	21	11.8	13	13.4	8	9.9	0.321
*DPYD* c.2846A>T	24	13.5	9	9.3	15	18.5	
*DPYD* c.1236G>A (HapB3)	76	42.7	38	39.2	38	46.9	
Compound heterozygous *DPYD* genotypes							
*DPYD* c.[1905+1G>A];[1679T>G]	1	0.6	1	1.0	0	0	
*DPYD* c.[1905+1G>A];[1236G>A]	1	0.6	1	1.0	0	0	
*UGT1A1* genotypes^b^							
*UGT1A1* *28/*28	54	30.3	35	36.1	19	23.5	0.307
*UGT1A1* *28/*6	1	0.6	1	1.0	0	0	
*UGT1A1* *28/*37	1	0.6	0	0	1	1.2	
5‐FU‐based regimen							
5‐FU + IRI	60	33.7	43	44.3	17	21.0	0.027
5‐FU + Pt	31	17.4	18	18.6	13	16.0	
5‐FU triplets	20	11.2	7	7.2	13	16.0	
Others^c^	12	6.7	6	6.2	6	7.4	
Capecitabine‐based regimen							
Cape mono	13	7.3	6	6.2	7	8.6	0.655
Cape + Pt	15	8.4	8	8.2	7	8.6	
Cape + RT	21	11.8	7	7.2	14	17.3	
Others^d^	3	1.7	1	1.0	2	2.5	
Irinotecan monotherapy	3	1.7	1	1.0	2	2.5	—
Setting							
Metastatic	103	57.9	57	58.8	46	56.8	
Neo‐adjuvant	33	18.5	12	12.4	21	25.9	0.078
Adjuvant	42	23.6	28	28.9	14	17.3	

Abbreviations: 5‐FU triplets, 5‐fluoruracil combined with two other chemotherapy agents; 5‐FU + IRI, 5‐fluorouracil combined with irinotecan; 5‐FU + Pt, 5‐Fluoruracil combined with platinum agent; Cape mono, capecitabine monotherapy; Cape + Pt, capecitabine combined with platinum agent; Cape + RT, capecitabine combined with radiotherapy; IQR, interquartile range; *N*, number of patients.

* *p* value was determined using Fisher's exact test or the Chi‐square test.

^a^ Two patients, one from the genotype‐driven group and one from the standard group, were Hispanic; one patient from the standard group was Indian; one patient from the genotype‐driven group was African; and three patients of unknown ancestry were in the standard group.

^b^ one patient in the standard group carried clinically relevant variants in both UGT1A1 (*28/*28) and DPYD (c.1236GA) and was included in both genotype categories.

^c^ other treatment regimens: 5‐FU monotherapy (*n* = 2); 5‐FU combined with panitumumab (*n* = 1); 5‐FU combined with cetuximab (*n* = 1); 5‐FU combined with oxaliplatin, irinotecan and bevacizumab (*n* = 7).

^d^ other treatment regimens: capecitabine combined with irinotecan (*n* = 1); capecitabine combined with temozolomide (*n* = 1); capecitabine with oxaliplatin and bevacizumab (*n* = 2).

Overall, 69.1% of patients carried any actionable *DPYD* genotype—most frequently the c.1236G>A (HapB3) variant in the heterozygous state—and 31.5% carried an actionable *UGT1A1* genotype, with no significant differences between groups. Only one patient (0.6%) in the standard‐of‐care group carried concomitant actionable *DPYD* and *UGT1A1* genotypes.

Patients received a range of fluoropyrimidine‐ and/or irinotecan‐based regimens. The only significant imbalance concerned certain 5‐FU–based chemotherapy regimens distribution, which were more frequently administered in the standard‐of‐care group (*p* = 0.027), while treatment setting was otherwise comparable (Table 1).

### 3.2 Treatment Outcomes (Toxicity, Treatment Modifications and Relative Dose Intensity) According to Specific Actionable Variants

Considering toxic effects over the entire treatment period, 71 out of 178 patients (39.9%) developed grade ≥ 3 toxicities related to fluoropyrimidine or irinotecan treatment (Table S1). Furthermore, 55 of 71 patients (77.5%) experienced at least one clinically relevant event (Table 2).

**TABLE 2:** Clinically relevant toxic events in the overall population, heterozygous *DPYD* variant carriers, and homozygous *UGT1A1* patients.

		Patients	Acute clinically relevant toxic events^a^			Overall clinically relevant toxic events^a^		
		*n*	*n* (%)	*p*	OR (95% CI)	*n* (%)	*p*	OR (95% CI)
Overall population	Standard‐of‐care	97	33 (34.0)	< 0.001*	1 [Reference]	42 (43.3)	< 0.001*	1 [Reference]
	Genotype‐driven	81	7 (8.6)		0.19 (0.08 to 0.47)*	13 (16.0)		0.27 (0.13 to 0.55)*
Heterozygous *DPYD* variants								
*DPYD* c.1905+1G>A	Standard‐of‐care	13	6 (46.2)			8 (61.5)		
	Genotype‐driven	8	0 (0.0)	0.046	—	0 (0.0)	0.007	—
*DPYD* c.2846A>T	Standard‐of‐care	9	5 (55.6)			6 (66.7)		
	Genotype‐driven	15	2 (13.3)	0.061	—	4 (26.7)	0.092	—
*DPYD* c.1236G>A (HapB3)	Standard‐of‐care	38	8 (21.1)			9 (23.7)		
	Genotype‐driven	38	3 (7.9)	0.191	—	5 (13.2)	0.375	—
*UGT1A1* genotype								
UGT1A1*28/*28	Standard‐of‐care	35	12 (34.3)			16 (45.7)		
	Genotype‐driven	19	2 (10.5)	0.102	—	4 (21.1)	0.086	—

*Note:* Only genotype groups with sufficient sample size were included as separate categories. Rare combinations (e.g., *DPYD* c.[1905+1G>A];[1679T>G], *DPYD* c.[1905+1G>A];[1236G>A], UGT1A1*28/*37, UGT1A1*28/*6) were not presented separately due to single‐patient counts but were included in the overall population analysis.

Abbreviation: *n*, number of patients.

^a^ Clinically relevant toxic events are defined as an hematological toxicity of grade 4 or higher or non‐hematological toxicity of grade 3 or higher. Acute toxicity was evaluated considering the first treatment cycle, while the overall one was assessed considering the whole treatment duration.

* Multivariate logistic regression adjusted for sex and age.

Clinically relevant toxicities were significantly more frequent in the standard‐of‐care group than in the genotype‐driven group, both during the first cycle (34.0% vs. 8.6%; adj OR, 0.19; 95% CI, 0.08–0.47; *p* < 0.001) and over the entire treatment period (adj OR, 0.27; 95% CI, 0.13–0.55, *p* < 0.001) (Table 2). Consistently, treatment modifications (i.e., dose reductions, delays, and interruptions) occurred in 86.6% (84 of 97) in the standard‐of‐care group compared with 45.7% (37 of 81) patients in the genotype‐driven group (adj OR, 0.15; 95% CI, 0.07–0.31; *p* < 0.001) (Table 3).

**TABLE 3:** Treatment modifications in the overall population, heterozygous *DPYD* variant carriers and homozygous *UGT1A1* patients.

		Patients	Any modification			Treatment delays			Dose reductions			Treatment interruptions		
		*n*	*n* (%)	OR (95% CI)	*p*	*n* (%)	OR (95% CI)	*p*	*n* (%)	OR (95% CI)	*p*	*n* (%)	OR (95% CI)	*p*
Overall population	Standard‐of‐care	97	84 (86.6)	1 [Reference]	< 0.001*	58 (59.8)	1 [Reference]	0.002*	32 (33.0)	1 [Reference]	< 0.001*	28 (28.9)	1 [Reference]	0.002*
	Genotype‐driven	81	37 (45.7)	0.15 (0.07 to 0.31)*		28 (34.6)	0.37 (0.20 to 0.70)*		4 (4.9)	0.10 (0.03 to 0.29)*		5 (6.2)	0.24 (0.10 to 0.58)*	
Heterozygous *DPYD* variants														
*DPYD* c.1905+1G>A	Standard‐of‐care	13	13 (100)	—	0.003	9 (69.2)	1 [Reference]	0.162	3 (23.0)	—	0.2016	6 (46.2)	—	0.046
	Genotype‐driven	8	3 (37.5)	—		3 (37.5)	0.26 (0.04 to 1.70)		0 (0.0)	—		0 (0.0)	—	
*DPYD* c.2846A>T	Standard‐of‐care	9	7 (77.8)	1 [Reference]	0.146	4 (44.4)	1 [Reference]	0.831	3 (33.3)	—	0.026	3 (33.3)	1 [Reference]	0.121
	Genotype‐driven	15	7 (46.6)	0.25 (0.04 to 1.62)		6 (40.0)	0.93 (0.16 to 4.43)		0 (0.0)	—		1 (6.7)	0.14 (0.01 to 1.67)	
*DPYD* c.1236G>A (HapB3)	Standard‐of‐care	38	30 (78.9)	1 [Reference]	0.001	18 (47.4)	1 [Reference]	0.161	12 (31.6)	—	< 0.001	10 (26.3)	1 [Reference]	0.085
	Genotype‐driven	38	16 (42.1)	0.19 (0.07 to 0.53)		12 (31.6)	0.51 (0.20 to 1.31)		0 (0.0)	—		4 (10.5)	0.33 (0.09 to 1.16)	
*UGT1A1* genotype														
UGT1A1*28/*28	Standard‐of‐care	35	31 (88.6)	1 [Reference]	0.006	27 (77.1)	1 [Reference]	0.005	14 (40.0)	1 [Reference]	0.104	7 (20.0)	—	0.037
	Genotype‐driven	19	10 (52.6)	0.14 (0.04 to 0.56)		7 (36.8)	0.17 (0.05 to 0.59)		4 (21.1)	0.31 (0.07 to 1.28)		0 (0.0)	—	

*Note:* Only genotype groups with sufficient sample size were included as separate categories. Rare combinations (e.g., *DPYD* c.[1905+1G>A];[1679T>G], *DPYD* c.[1905+1G>A];[1236G>A], UGT1A1*28/*37, UGT1A1*28/*6) were not presented separately due to single‐patient counts but were included in the overall population analysis.

Abbreviation: *n*, number of patients.

* Multivariate logistic regression adjusted for sex and age.

**FIGURE 2:**
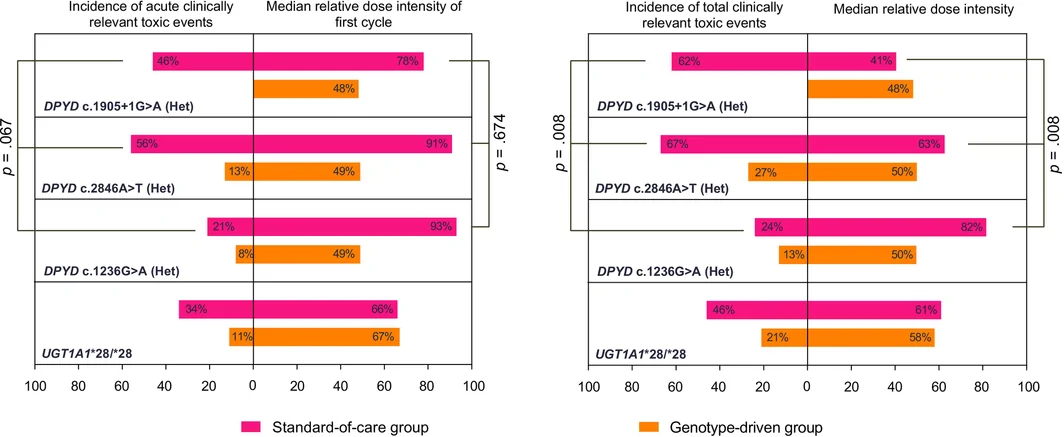
Incidence of acute and total clinically relevant toxicities and median relative dose intensity, stratified by genotype. The figure illustrates the impact of genotype‐specific dose adjustments on both toxicity occurrence and dosing intensity across the treatment course. *p* values were calculated using the Mann–Whitney *U* test or Fisher's exact test, as appropriate.

Given the variability observed across *DPYD* genotypes, a comparative analysis of toxicity incidence among the three *DPYD* variant carriers was conducted within the standard‐of‐care group. Overall clinically relevant toxicity rates in the standard‐of‐care group differed significantly across variants (*p* = 0.008, Fisher's exact), whereas differences in first‐cycle toxicity showed borderline statistical significance (*p* = 0.067, Fisher's exact) (Figure 2). Overall RDI in the standard‐of‐care group differed significantly across variants (*p* = 0.008, Mann–Whitney *U* test), whereas first‐cycle RDI showed no statistically significant difference (*p* = 0.674, Mann–Whitney *U* test) (Figure 2).

Patients in the standard‐of‐care group started the treatment at full doses according to the prescribed regimen, whereas those in the genotype‐driven dosing group began therapy at a reduced starting dose according to DPWG recommendation (Table 4). Trends of median RDI across treatment cycles in carriers of clinically relevant *DPYD* or *UGT1A1* variants are shown in Figure S1. The figure shows that patients receiving genotype‐driven dose reductions generally maintained stable RDI, while more variability was observed in patients receiving full starting doses. These findings should be interpreted with caution, as the number of patients contributing to each cycle decreased over time due to treatment discontinuation.

**TABLE 4:** Day 1 administered dose and relative dose intensity (RDI) in the overall population, heterozygous *DPYD* variant carriers and homozygous *UGT1A1* patients. Data are presented as median values with interquartile ranges (IQR).

		Patients	% Dose administered (Day 1), median (IQR)	First cycle RDI%		Overall RDI%	
		*n*		Median (IQR)	*p*	Median (IQR)	*p*
Heterozygous *DPYD* variants							
*DPYD* c.1905+1G>A	Standard‐of‐care	13	100 (100–100)	78 (58–100)	0.014	41 (13–62)	0.425
	Genotype‐driven	8	50 (49–50)	48 (40–50)		48 (46–50)	
*DPYD* c.2846A>T	Standard‐of‐care	9	100 (99–100)	91 (80–96)	0.002	63 (54–80)	0.007
	Genotype‐driven	15	50 (50–53)	49 (41–51)		50 (43–50)	
*DPYD* c.1236G>A (HapB3)	Standard‐of‐care	38	100 (98–100)	93 (76–100)	< 0.001	82 (63–92)	< 0.001
	Genotype‐driven	38	50 (50–53)	49 (41–52)		50 (43–55)	
*UGT1A1* genotype							
UGT1A1*28/*28	Standard‐of‐care	35	99 (88–100)	66 (52–80)	0.208	61 (41–76)	0.906
	Genotype‐driven	19	70 (68–74)	67 (52–70)		58 (50–69)	

*Note:* Only genotype groups with sufficient sample size were included as separate categories. Rare combinations (e.g., *DPYD* c.[1905+1G>A];[1679T>G], *DPYD* c.[1905+1G>A];[1236G>A], UGT1A1*28/*37, UGT1A1*28/*6) were not presented separately due to single‐patient counts but were included in the overall population analysis.

Abbreviation: *n*, number of patients.

#### 3.2.1 Heterozygous *DPYD* c.1905+1G>A (*2A) Carriers

As shown in Table 2 and Figure 2, among heterozygous carriers of *DPYD* c.1905+1G>A, clinically relevant toxicities occurred exclusively in the standard‐of‐care group both during the first cycle (46.2% vs. 0% of patients; OR not evaluable, *p* = 0.046) and over the entire treatment period (61.5% vs. 0% of patients; OR not evaluable, *p* = 0.007).

With regard to RDI, these patients showed a marked reduction over time in the standard‐of‐care group, with median RDI declining from 78% (IQR 58–100) after the first cycle to 41% (IQR 13–62) over the entire treatment period (Table 4, Figure 2). In contrast, patients in the genotype‐driven group had an RDI of 48% (IQR 40–50) after the first cycle, which was maintained over the entire treatment period (RDI 48%, IQR 46–50). Overall RDI was higher, although not significantly, in the genotype‐driven group (median 48%, IQR 46–50) compared with the standard‐of‐care group (median 41%, IQR 13–62) (*p* = 0.425).

Consistently, early treatment discontinuation occurred in 6 of 13 patients (46.2%) in the standard‐of‐care group, while no discontinuations were observed among patients in the genotype‐driven group (OR not evaluable, *p* = 0.046). Full data are provided in Table 3.

#### 3.2.2 Heterozygous *DPYD* c.2846A>T Carriers

Heterozygous carriers of *DPYD* c.2846A>T in the standard‐of‐care group experienced higher rates of clinically relevant toxicities compared with those in the genotype‐driven group. Acute clinically relevant toxicities occurred in 55.6% of patients in the standard‐of‐care group versus 13.3% in the genotype‐driven group (OR not evaluable, *p* = 0.061), with a median RDI of 91% (IQR 80–96) versus 49% (IQR 41–51), respectively.

Over the entire treatment course, patients in the standard‐of‐care group received a median RDI of 63% (IQR 54–80), compared with 50% (IQR 43–50) in the genotype‐driven group, at the cost of higher toxicity rates (66.7% vs. 26.7%; OR not evaluable, *p* = 0.092) (Table 2, Table 4, Figure 2).

Regarding treatment modifications, dose reductions occurred in 33.3% of carriers in the standard‐of‐care group, whereas no dose reduction was observed in the genotype‐driven group (OR not evaluable, *p* = 0.026) (Table 3).

#### 3.2.3 Heterozygous *DPYD* c.1236G>A (HapB3) Carriers

No significant difference in the risk of clinically relevant toxicity was observed in heterozygous carriers of *DPYD* c.1236G>A (HapB3) in the standard‐of‐care group and those in the genotype‐driven group (Table 2, Figure 2). Acute clinically relevant toxicities occurred in 21.1% of patients in the standard‐of‐care group versus 7.9% in the genotype‐driven group (OR not evaluable, *p* = 0.191), while overall clinically relevant toxicities were observed in 23.7% and 13.2% of patients, respectively (OR not evaluable, *p* = 0.375).

In terms of dose intensity, carriers showed a moderate reduction over time in the standard‐of‐care group, with a median RDI of 93% (IQR 76–100) after the first cycle and 82% (IQR 63–92) over the entire treatment period (Table 4, Figure 2). In contrast, patients in the genotype‐driven group had an RDI of 49% (IQR 41–52) after the first cycle and an RDI of 50% (IQR 43–55) over the entire treatment period. Overall RDI in the genotype‐driven group (median 50%, IQR 43–55) was lower compared with the standard‐of‐care group (median 82%, IQR 63–92) (*p* < 0.001).

As shown in Table 3, dose reductions were observed exclusively in the standard‐of‐care group, affecting 31.6% of carriers, whereas no dose reductions occurred in the genotype‐driven group (OR not evaluable, *p* < 0.001). Among the 12 patients who required dose reductions, 10 were reduced to 75% of the initial dose and 2%–50%.

Notably, within the first five treatment cycles, dose escalation was feasible in 23.7% (9 out of 38) of patients in the genotype‐driven group, reaching up to 75% (IQR 66–75) of the standard dose without clinically relevant toxicity. Some patients approached doses close to 100% of the standard dose without developing clinically relevant toxicity.

#### 3.2.4 *UGT1A1* *28 Homozygous Carriers

Homozygous carriers of UGT1A1*28 in the standard‐of‐care group experienced higher rates of clinically relevant toxicities compared with those in the genotype‐driven group (Table 2, Figure 2). Acute clinically relevant toxicities occurred in 34.3% of patients in the standard‐of‐care group versus 10.5% in the genotype‐driven group (OR not evaluable, *p* = 0.102), while overall clinically relevant toxicities were observed in 45.7% and 21.1% of patients, respectively (OR not evaluable, *p* = 0.086).

In terms of dose intensity, carriers showed a moderate reduction over time in the standard‐of‐care group, with median RDI declining from 66% (IQR 52–80) after the first cycle to 61% (IQR 41–76) over the entire treatment period (Table 4). In contrast, patients in the genotype‐driven group had an RDI of 67% (IQR 52–70) after the first cycle and an RDI of 58% (IQR 50–69) over the entire treatment period. Overall RDI was not significantly lower in the genotype‐driven group than in the standard‐of‐care group (58% vs. 61%; *p* = 0.906).

Regarding treatment modifications, 27 out of 35 patients in the standard‐of‐care group (77.1%) experienced treatment delays, compared with 7 out of 18 (36.8%) in the genotype‐driven group (adj OR, 0.17; 95% CI, 0.05–0.59; *p* = 0.005). Furthermore, 7 out of 35 patients (20.0%) in the standard‐of‐care group discontinued treatment prematurely, whereas no patients in the genotype‐driven group experienced early treatment interruption. Full data are provided in Table 3.

Other *UGT1A1* poor metabolizers (i.e., one patient with a UGT1A1*28/*6 genotype in the standard‐of‐care group and one patient with a UGT1A1*28/*37 in the genotype‐driven group) were not analyzed separately since the numbers were too low. Clinical outcomes of these patients are reported in the section below.

### 3.3 Treatment Outcomes in *DPYD* and *UGT1A1* Compound Heterozygous Carriers

Two patients with compound heterozygous *DPYD* variants received fluoropyrimidine‐based chemotherapy: one carrying c.1905+1G>A and c.1679T>G, and one with c.1905+1G>A and c.1236G>A (HapB3). Both patients were treated at full‐dose chemotherapy and experienced severe fluoropyrimidine‐related non‐hematologic toxicity. The patient with c.1905+1G>A and c.1236G>A discontinued treatment after one cycle due to grade 3 diarrhea, resulting in an overall RDI of 8.0%. In contrast, the patient carrying both c.1905+1G>A and c.1679T>G died after one cycle of full‐dose chemotherapy due to severe 5‐FU‐related diarrhea and stomatitis, achieving an overall RDI of 12.5%.

Two patients with compound heterozygous *UGT1A1* variants were also identified among the study population. The first patient, with UGT1A1*28/*6 genotype, assigned to the standard‐of‐care group, developed grade 3 nausea during the treatment, leading to dose reduction and an overall RDI of 75%. In contrast, the patient with UGT1A1*28/*37 genotype, in the genotype‐driven group, did not experience any toxicities throughout the treatment and had an overall RDI of 62%.

## 4 Discussion

This study reflects the real‐world implementation of pretreatment *DPYD* and *UGT1A1* testing and genotype‐driven dosing for fluoropyrimidines and irinotecan in an unselected population of cancer patients carrying actionable genotypes in either *DPYD* or *UGT1A1* and across multiple clinical settings. Although previous studies have consistently demonstrated that pretreatment pharmacogenetic testing with recommended genetic panels mitigates severe fluoropyrimidine‐ and irinotecan‐related toxicity without compromising overall treatment outcomes or intensity [13, 14, 15], less is known about the variant‐specific effects in a real‐world practice context.

Leveraging a large, prospectively accrued patient cohort, we compared patients treated at standard full doses with post hoc genotyping to those receiving pretreatment genotyping and DPWG‐driven dose reductions [10, 11]. Our findings provide, for the first time, real‐world evidence that the clinical impact of genotype‐driven dosing is strongly variant‐dependent, revealing a clear heterogeneity among *DPYD* variants in terms of toxicity risk and treatment intensity.

Functional in vitro studies have demonstrated that actionable *DPYD* variants differ substantially in their impact on DPD activity, with c.1905+1G>A and c.1679T>G causing severe deficiency, and c.2846A>T and c.1236G>A (HapB3) associated with more moderate reductions [23, 24, 25]. Consistently, in a pretreatment phenotyping subgroup analysis, Henricks et al. [14] reported that *DPYD* c.1236G>A (HapB3) and c.2846A>T carriers exhibited approximately 20% and 35% reductions in DPD enzyme activity, respectively, whereas c.1905+1G>A and c.1679T>G were associated with more pronounced activity decreases of about 45% and 60%. In this context, concerns have been raised regarding the application of a uniform 50% starting dose reduction for all carriers of actionable *DPYD* variants [18, 19, 26, 27]. When RDI is considered as a proxy for drug exposure, a uniform upfront reduction may not be appropriate for all genotypes and could lead to unnecessary underexposure in carriers of milder phenotypes, such as c.1236G>A (HapB3) [15]. Recently, Ratain and O'Donnell have commented that fixed, uniform dosing strategies fail to capture substantial interindividual variability in drug exposure and tolerability, with particular emphasis on capecitabine‐based regimens [27]. These observations underscore the limitations of applying the same dose reduction to all patients irrespective of genotype. In this context, our real‐world findings suggest that pharmacogenetic stratification provides a structured framework to guide treatment, allowing variant‐specific initial dose reductions and subsequent adjustments. By distinguishing between high‐, intermediate‐, and lower‐impact *DPYD* variants, genotype‐driven dosing refines therapy to preserve both safety and adequate drug exposure without challenging the overall validity of current pharmacogenetic guidelines.

In the present study, heterozygous carriers of *DPYD* c.1905+1G>A treated according to standard‐of‐care represented the subgroup at highest clinical risk both after the first cycle and over the entire treatment course. These patients, however, achieved an overall RDI lower than that observed in the genotype‐driven group (41% vs. 48%, respectively), reflecting the substantial downstream impact of toxicity‐driven dose interruptions, reductions, and early discontinuations in patients treated without prior genotyping [28, 29]. This evidence supports a 50% upfront dose reduction approach in carriers of *DPYD* c.1905+1G>A [14, 17, 18, 28, 29], which has been shown to be effective in reducing drug‐related toxicity while preserving the intensity of the chemotherapy [14, 17, 18].

Heterozygous carriers of *DPYD* c.2846A>T treated at full dose achieved a slightly higher overall RDI compared with those receiving genotype‐driven dosing (63% vs. 50%). This may appear to be in contrast with our previous findings reporting a lower RDI in carriers of *DPYD* variants undergoing standard‐of‐care fluoropyrimidine dosing [13]. However, it should be noted that patients in the genotype‐driven group were treated more recently (2019–2025), whereas those in the standard‐of‐care group were treated in an earlier period (2002–2018). In earlier treatment eras, limited therapeutic options—particularly in the metastatic setting—may have discouraged treatment interruption or modification despite toxicity, potentially biasing the comparison toward an underestimation of the true impact of genotype‐driven dosing on treatment exposure. This temporal effect is likely more pronounced for *DPYD* c.2846A>T, whereas in very high‐risk subgroups such as carriers of *DPYD* c.1905+1G>A and homozygous carriers of UGT1A1*28, severe toxicity made dose reductions and treatment interruptions unavoidable even in earlier periods.

Indeed, heterozygous carriers of *DPYD* c.2846A>T in the standard‐of‐care group received a slightly higher RDI but at the cost of a markedly higher toxicity burden, with frequent treatment delays and early discontinuations. Specifically, 66.7% of patients in the standard‐of‐care group experienced at least one clinically relevant toxicity compared with 26.7% in the genotype‐driven group. The more moderate effect on treatment adjustments compared with *DPYD* c.1905+1G>A is consistent with prior studies showing that *DPYD* c.2846A>T confers an intermediate reduction in DPD activity, translating into a clinically relevant but variable toxicity risk [14, 18, 30, 31]. In this context, the observed reduction in overall RDI in genotype‐driven patients occurred alongside a substantially lower toxicity burden, potentially reflecting an acceptable trade‐off between treatment exposure and safety in this intermediate‐risk subgroup.

In contrast, heterozygous carriers of *DPYD* c.1236G>A (HapB3) displayed a distinct pattern. In line with a previous report [31], under standard‐of‐care treatment, these patients experienced severe toxicity in 23.7% of the cases, which was markedly lower than that observed for the other two *DPYD* variants (61.5%–66.7%) and comparable to the 20%–30% incidence consistently reported across multiple studies in *DPYD* wild‐type populations [14, 26, 32]. This observation aligns with biochemical and clinical evidence indicating that *DPYD* c.1236G>A (HapB3) is associated with a relatively mild reduction in DPD activity (~20%), substantially lower than that observed for *DPYD* c.1905+1G>A or *DPYD* c.2846A>T [14, 23, 24]. This limited clinical impact is further corroborated by the recent work of Boige et al. [33], where *DPYD* c.1129–5923C>G (HapB3 causative variant) was excluded from the final predictive model as it was not consistently selected by the Bolasso algorithm and lacked a significant association with G4‐5 toxicity.

Consistent with the low incidence of clinically relevant toxicity observed in heterozygous carriers of *DPYD* c.1236G>A (HapB3) under standard‐of‐care dosing, overall RDI remained relatively unchanged over time, and most carriers (68.4%, data not shown) achieved an overall fluoropyrimidine RDI greater than 50% without developing clinically relevant toxicities. Notably, only a minority of patients (31.6%) in the standard‐of‐care group required a dose reduction during treatment due to fluoropyrimidine intolerance. In most cases (10 out of 12, 83.3%), a 25% reduction was sufficient to control toxicity for the subsequent cycles. Regardless, genotype‐guided dosing resulted in a marked reduction in RDI without a clear reduction in toxicity. These findings align with previous studies where the average tolerated dose was found to be between 71% and 74% [14, 18]; however, methodological differences in RDI calculation limit direct comparability. Moreover, consistent with observations by Peeters et al., 23.7% of genotype‐driven c.1236G>A (HapB3) heterozygous carriers in our cohort safely underwent dose escalation to a median of ~75% of the standard dose (IQR 66%–75%), with some patients approaching full‐dose exposure without experiencing clinically relevant toxicity. Nevertheless, in line with numerous previous studies [14, 18, 31, 34], most patients were not dose‐escalated despite minimal toxicity, reflecting persistent clinical caution in dose individualization for *DPYD* variants.

Considering the relatively modest functional impact of the HapB3 tagging variant, these data suggest that uniform genotype‐based dosing strategies may not optimally capture the heterogeneity of *DPYD* variant effects. Carriers of *DPYD* c.1236G>A (HapB3) could potentially benefit from more flexible or adaptive dosing strategies to avoid underexposure in patients with relatively preserved fluoropyrimidine tolerance. This is also consistent with previous studies suggesting a higher risk of treatment failure in these patients treated at reduced doses [13]. At the same time, the observed interindividual variability underscores the importance of careful patient monitoring during treatment and throughout the dose‐adjustment phase [27].

In homozygous UGT1A1*28 carriers, upfront genotype‐driven dose reductions substantially improved the toxicity profile during the first cycle, without significantly compromising RDI. In the standard‐of‐care group, RDI rapidly declined and became comparable to that of the genotype‐driven group (66% vs. 67%, respectively) by the end of the first cycle, highlighting that irinotecan‐related toxicity exerts its greatest impact during the early phases of treatment, often leading to early dose reductions or delays. Moreover, consistent with our previous findings [17], the incidence of clinically relevant toxicities in the genotype‐driven group was reduced by more than 50%. This underlines how, in UGT1A1*28 homozygous carriers, upfront dose reductions effectively protected patients from severe toxicity while preserving overall drug exposure.

In this study, we also included a case of fatal toxicity after a single cycle of standard‐of‐care fluoropyrimidine therapy in a carrier of compound heterozygous genotype (c.1905+1G>A and c.1679T>G). In addition, another patient, compound heterozygous for c.1905+1G>A and c.1236G>A (HapB3), developed grade 3 diarrhea and discontinued therapy after a single cycle. This observation aligns with previously reported cases of compound heterozygous *DPYD* carriers, in which standard‐dose fluoropyrimidine therapy frequently resulted in acute severe to life‐threatening toxicity [35]. These findings confirm that carriers of a compound heterozygous *DPYD* genotype, currently corresponding to a gene activity score of 0–0.5, according to the Clinical Pharmacogenetics Implementation Consortium guidelines (CPIC), or PHENO according to DPWG guidelines, need to avoid fluoropyrimidine treatment unless a phenotyping assay is available to establish residual DPD activity [10, 36, 37].

Collectively, these findings reveal a clear hierarchy of clinical impact among *DPYD* and *UGT1A1* genotypes. Heterozygous carriers of *DPYD* c.1905+1G>A and homozygous carriers of UGT1A1*28 represent high‐risk patients for which an upfront dose reduction of 50% and 30%, respectively, provides clear benefits in terms of both treatment safety and intensity.

In contrast, *DPYD* c.1236G>A (HapB3) appears to have a lower clinical impact, and current recommendations may not fully reflect the variability in its clinical impact. In this subgroup, a more moderate initial dose reduction or an early dose‐escalation strategy may be sufficient to balance treatment safety and adequate drug exposure [38], as recommended by current Italian and French pharmacogenetic guidelines [39, 40]. However, these findings should be interpreted with caution given the limited sample size and the lack of statistically significant differences in toxicity outcomes. Conversely, for heterozygous carriers of *DPYD* c.2846A>T, a 50% upfront dose reduction still appears appropriate, as the slightly reduced overall RDI associated with genotype‐driven dosing was associated with a substantially improved toxicity profile.

The main limitation of this study is the non‐contemporaneous nature of the two cohorts (2002–2018 vs. 2018–2025), which may introduce a temporal bias. While baseline characteristics were balanced, the recent cohort reflects an evolution in clinical practice, characterized by a higher prevalence of intensified regimens and updated supportive care protocols. To account for such heterogeneity, we conducted a sensitivity analysis on a subset of 47 patients treated between 2017 and 2020, a period encompassing the transition from standard‐of‐care to genotype‐driven practice. Despite the limited sample size, this analysis showed that the effect of the intervention on RDI and toxicity occurrence by variant is substantially maintained (data not shown). Moreover, it should be noted that the relatively large and unselected cohort of patients carrying clinically relevant *DPYD* and *UGT1A1* variants reflects real‐world clinical practice and enhances the generalizability of the findings.

As a further study limitation, the non‐blinded case–control design may have introduced a potential bias. When genetic results are available, clinicians tend to adopt a more cautious and standardized approach, applying dose reductions in accordance with the identified pharmacogenetic risk. In contrast, in the absence of genetic information, decisions rely solely on clinical judgment and best practice, and individual clinicians may be more inclined to maintain full doses or avoid treatment delays. This behavioral difference may further contribute to the attenuated contrast in dose intensity observed between case and control groups.

It is also worth noting that genotyping was performed for the HapB3 tagging single nucleotide polymorphism rs56038477 (c.1236G>A) rather than the causal intronic variant rs75017182 (c.1129‐5923C>G). Although c.1236G>A is commonly used as a surrogate marker, recent evidence suggests that it is not always in complete linkage disequilibrium with the causal variant. Therefore, the inference of reduced DPD enzyme activity based solely on c.1236G>A may not fully capture all patients with decreased DPD function.

## 5 Conclusion

In conclusion, in our cohort genotype‐driven dosing based on *DPYD* and *UGT1A1* variants appears to enhance treatment safety without substantially compromising RDI, thereby supporting its effectiveness and feasibility in routine oncology practice.

Significant upfront dose reductions were clearly beneficial for patients carrying high‐risk *DPYD* variants, such as c.1905+1G>A, and homozygous carriers of UGT1A1*28. A 50% reduction also remained justified for the intermediate‐risk *DPYD* c.2846A>T variant. In contrast, applying uniform dose reductions to heterozygous carriers of the *DPYD* c.1236G>A (HapB3) did not provide a clear safety advantage and was associated with reduced treatment exposure. These findings suggest that a uniform dose‐reduction strategy may not fully capture the variability of *DPYD* c.1236G>A (HapB3) carriers and could potentially result in underexposure [18, 19]. Refined, variant‐specific dosing strategies, such as more moderate initial reductions or adaptive dose escalation protocols, may therefore be considered to optimize the balance between safety and therapeutic intensity in pharmacogenetically driven oncology. However, these findings require confirmation in larger prospective studies.

## Author Contributions

**Martina Gambron:** data curation, formal analysis, investigation, methodology, software, visualization, writing – original draft, writing – review and editing. **Elena Peruzzi:** data curation, formal analysis, investigation, methodology, writing – original draft. **Samantha Perfler:** software. **Marcella Montico:** formal analysis. **Riccardo Cecchin:** data curation. **Elena De Mattia:** data curation. **Michele Spina:** resources, writing – review and editing. **Camilla Lisanti:** resources, writing – review and editing. **Serena Corsetti:** resources, writing – review and editing. **Luisa Foltran:** resources, writing – review and editing. **Fabio Puglisi:** resources, writing – review and editing. **Rossana Roncato:** methodology, supervision, visualization, writing – original draft, writing – review and editing. **Erika Cecchin:** conceptualization, funding acquisition, project administration, resources, supervision, writing – original draft, writing – review and editing, validation.

## Funding

This work was supported by the Italian Ministry of Health (Ricerca Corrente).

## Ethics Statement

The study was approved by the local Ethics Committee.

## Consent

Signed informed consent was obtained from all patients for the use of their data for research purposes.

## Conflicts of Interest

C.L. declares honoraria for advisory boards from Daiichi Sankyo, travel grants from MSD and Eli Lilly and honoraria for activities as a speaker from Novartis. F.P. reports honoraria for advisory boards, activities as a speaker, travel grants, research grants from Astrazeneca, Bayer, Bristol Myers Squibb, Daiichi Sankyo, Eli Lilly, Exact Sciences, Gilead, Italfarmaco, Menarini, MSD, Novartis, Pfizer, Roche; Research funding from Astrazeneca, Eisai, Roche. The remaining authors declare no conflicts of interest.

## Supporting information


**Table S1:** Toxicity outcomes in fluoropyrimidines and irinotecan‐based treatment.
**Figure S1:** Overview of median relative dose intensity cycle‐by‐cycle in carriers of clinically relevant *DPYD* and *UGT1A1* variants. (A) Heterozygous *DPYD* c.1905+1G>A carriers; (B) Heterozygous *DPYD* c.2846A>T carriers; (C) Heterozygous *DPYD* c.1236G>A (HapB3) carriers; (D) Patients with UGT1A1*28/*28 genotype.

## Data Availability

Data supporting the findings of this study are available within the article. Additional information is available from the corresponding author upon reasonable request.
