# Supplementary material for: DPYD and UGT1A1 Genotype‐Based Dosing for Fluoropyrimidines and Irinotecan Chemotherapy: Variant‐Specific Impact on Treatment Intensity and Toxicity
Source: Int J Cancer. 2026 Jun 9;159(10):2512–24. doi: 10.1002/ijc.70580 (PMC13595455; doi:10.1002/ijc.70580)
Supplement: Supplementary file 1 [file IJC-159-2512-s001.pdf]

Supplementary material

MANUSCRIPT:

***DPYD* and *UGT1A1* genotype-based dosing for fluoropyrimidines and irinotecan chemotherapy: variant-specific impact on treatment intensity and toxicity**

Martina Gambron, Elena Peruzzi, Samantha Perfler, Marcella Montico, Riccardo Cecchin, Elena De Mattia, Michele Spina, Camilla Lisanti, Serena Corsetti, Luisa Foltran, Fabio Puglisi, Rossana Roncato, Erika Cecchin

TABLE OF CONTENTS:

Supplementary Table

- **Table S1.** Toxicity outcomes in fluoropyrimidines and irinotecan-based treatment.

Supplementary Figure

- **Figure S1.** Overview of median relative dose intensity cycle-by-cycle in carriers of clinically relevant *DPYD* and *UGT1A1* variants. (A) Heterozygous *DPYD* c.1905+1G>A carriers; (B) Heterozygous *DPYD* c.2846A>T carriers; (C) Heterozygous *DPYD* c.1236G>A (HapB3) carriers; (D) Patients with *UGT1A1**28/*28 genotype.

Table S1. **Toxicity outcomes in fluoropyrimidines and irinotecan-based treatment.**

	**Standard-of-care** (n=97)		**Genotype-driven** (n=81)	
	n	%	n	%
**Acute toxicity**				
Any type of toxicity ≥ G3	37	38.1	8	9.9
Non-hematologic toxicity ≥ G3	27	27.8	7	8.6
Hematologic toxicity ≥ G3	20	20.6	3	3.7
**Overall toxicity**				
Any type of toxicity ≥ G3	53	54.6	18	22.2
Non-hematologic toxicity ≥ G3	36	37.1	13	16
Hematologic toxicity ≥ G3	34	35.1	8	9.9

Abbreviation: n, number of patients

Figure S1. Overview of median relative dose intensity cycle-by-cycle in carriers of clinically relevant *DPYD* and *UGT1A1* variants. (A) Heterozygous *DPYD* c.1905+1G>A carriers; (B) Heterozygous *DPYD* c.2846A>T carriers; (C) Heterozygous *DPYD* c.1236G>A (HapB3) carriers; (D) Patients with *UGT1A128/*28 genotype.**

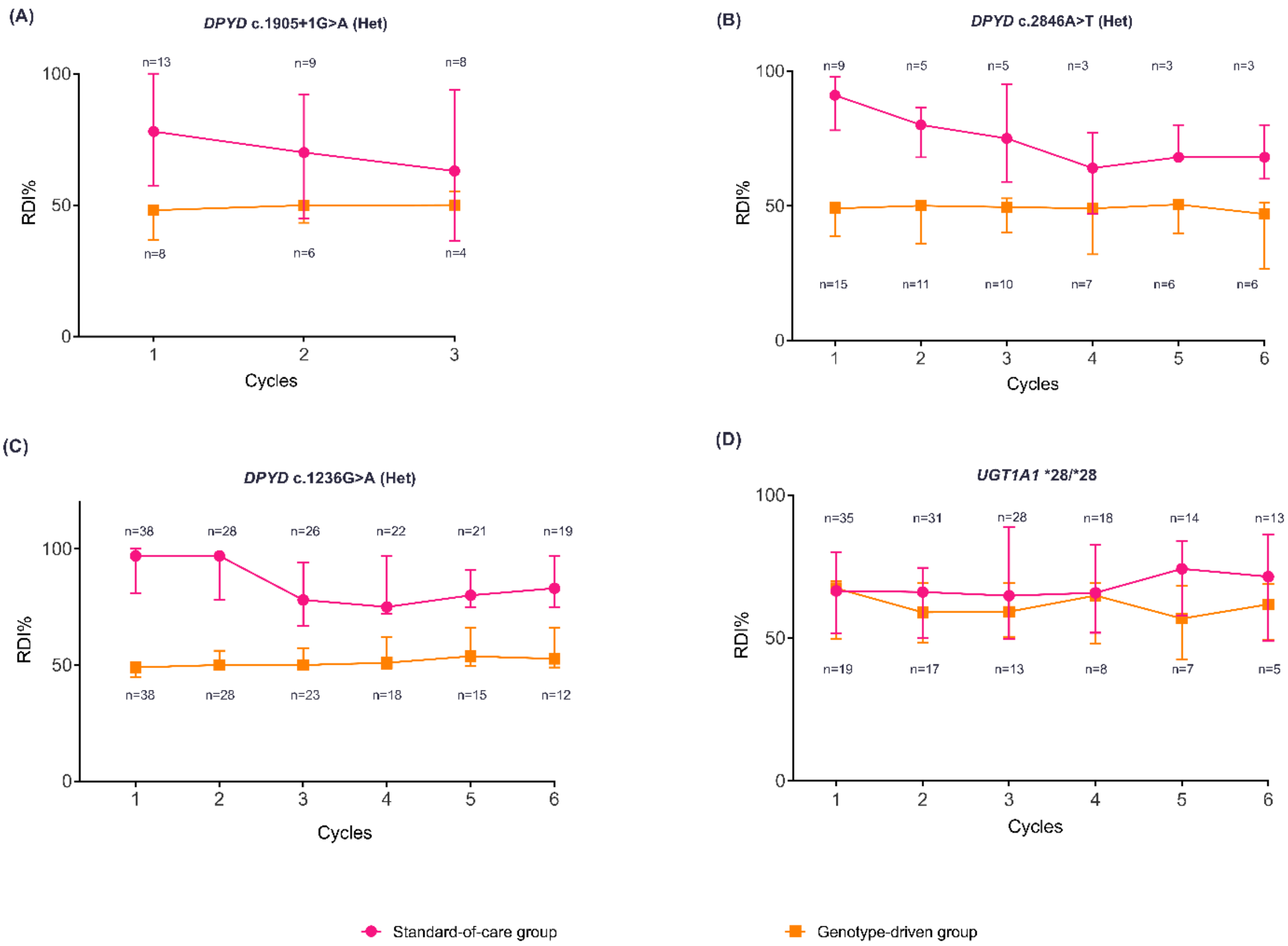

Abbreviations: RDI, relative dose intensity.
